# Preoperative Snack Prescription: A Single-Centre Experience in Optimising Preoperative Fasting Time and Enhancing Guideline Adherence

**DOI:** 10.7759/cureus.46271

**Published:** 2023-09-30

**Authors:** Steven H.S. Tan, Mohamed S M Elshikhawoda, Sohaib Jararaa, Che-Pin Cheung, Hassan Jararah

**Affiliations:** 1 Trauma and Orthopaedics, University Hospital Llandough, Penarth, GBR; 2 Vascular Surgery, Glan Clwyd Hospital, Rhyl, GBR; 3 Trauma and Orthopaedics, Bronglais Hospital, Aberystwyth, GBR

**Keywords:** preoperative snack, urgent surgeries, elective surgical procedures, nice guidelines, guideline, patient safety and quality improvement, general anaesthesia, perioperatvie pulmonary aspiration, preoperative fasting, pre-operative management

## Abstract

Objectives

Preoperative fasting plays a pivotal role in adequately preparing patients for anaesthesia and surgical procedures. However, it is imperative to consider not only the medical aspects but also patients' overall comfort, as this can significantly contribute to improved surgical outcome. The primary objective of this quality improvement project (QIP) is to provide healthcare professionals, including anaesthetists, surgeons, nurses, and stakeholders with information regarding insights required to embrace the concept of preoperative snack prescription as a strategy for enhancing patient-centred care.

Methods

This QIP was conducted in the vascular surgery department of a district general hospital in Wales, United Kingdom. A prospective analysis was conducted in two cycles, i.e., the pre-intervention group (PrIG) and post-intervention group (PoIG), with preoperative snacks such as biscuits, chips, or cakes, being prescribed to the PoIG. A total of 40 patients who met the inclusion criteria were enrolled in this study, with 20 patients participating in each cycle. The timing of preoperative meals, i.e., the closest preoperative breakfast, lunch, or dinner, preoperative snacks (for the PoIG), anaesthesia commencement, and surgical commencement were collected. Data analysis was performed using IBM SPSS Statistics for Windows, Version 26.0 (Released 2019; IBM Corp., Armonk, New York, United States), in conjunction with Microsoft Excel (Microsoft Corporation, Redmond, Washington, United States).

Results

In our QIP, the PrIG and PoIG comprised 40% (8 out of 20) and 35% (7 out of 20) female patients, respectively, with mean ages of 74 years (range, 61-86 years) and 61.3 years (range, 36-81 years). Within the PrIG, the mean duration from the preoperative meal to anaesthesia and surgery commencement was 17.8 hours (range, 14.6-22.5 hours) and 18.5 hours (range, 16.0-23.3 hours), respectively. In the PoIG, following the initiation of preoperative snack prescription, the mean time intervals between preoperative snack prescription and anaesthesia and surgery commencement were 10.9 hours (range, 6.5-16.0 hours) and 12.0 hours (range, 7.5-16.5 hours), respectively.

Conclusions

In summary, our QIP has successfully integrated preoperative snack prescription into the local hospital's preoperative care policy, prioritising the balance between patient safety and comfort. Based on our single-centre experience, we observed a significant reduction in the time interval between preoperative fasting and the initiation of anaesthesia, decreasing from 18.3 hours to 10.9 hours post-implementation of preoperative snacks. This QIP holds relevance for healthcare professionals as it underscores the benefits of shorter fasting periods, which contribute to heightened patient satisfaction and comfort.

## Introduction

Background

In the field of medicine, the pursuit of patient safety and enhanced surgical outcomes stands as a paramount concern. As healthcare practices continually evolve, so too does our understanding of the preoperative phase, a critical juncture where meticulous planning and preparation can significantly influence the course of surgery and subsequent recovery. Among the essential considerations during this phase, preoperative fasting has garnered significant attention.

Fasting before surgery is considered a standard policy aimed at minimising the risk of aspiration during the anaesthesia commencement [1]. The prevailing belief was that an empty stomach reduced the likelihood of regurgitated gastric contents entering the airways, thereby mitigating the potential for pulmonary complications [2]. Patients were often instructed to abstain from food for a prescribed duration before surgery, contributing to discomfort, anxiety, and thirst [3]. However, recent scientific advances and a growing body of evidence have challenged the necessity of prolonged fasting, leading to a re-evaluation of this practice [2].

Nowhere is this commitment more evident than in preoperative care, where innovative strategies continue to reshape long-standing traditions. The recommendation for preoperative snacks has become a compelling approach to improve both the patient experience and surgical preparedness. Presently, a conspicuous gap exists in the body of research dedicated to preoperative nutrition, with scant reference to the subject of preoperative snack prescription.

This quality improvement project (QIP) aims to explore the pivotal role that preoperative snack prescription plays in revolutionising preoperative fasting policy. We focused on the evolving understanding of the risks and benefits associated with fasting and how preoperative snacks can mitigate some of these concerns. Furthermore, we examine the impact of this innovative approach on patient comfort, ultimately improving surgical outcomes.

By fostering a holistic approach to patient-centred care while adhering to evidence-based principles, we hope to bridge the gap between established practices and the evolving landscape of surgical medicine, ultimately enhancing the safety and well-being of our patients.

Objectives

The aim of this QIP is to provide healthcare professionals, including anaesthetists, surgeons, nurses, and stakeholders with the knowledge and insights necessary to adopt preoperative snack recommendations as a way to enhance patient-centred care. By redefining the role of fasting in the preoperative phase, we not only prioritize patient well-being and safety but also contribute to a more patient-friendly, evidence-based approach to surgical preparation that aligns with the evolving landscape of healthcare. With note, preoperative fasting in this QIP refers to abstaining from solid food as this serves as the main argument in peri-operative care, while multiple studies have already discussed preoperative fluid restriction and this will not be discussed in this QIP.

## Materials and methods

This QIP was conducted in the vascular surgery department at Glan Clwyd Hospital in Wales, United Kingdom, from May 1, 2023, to August 31, 2023. The project was conducted in two cycles: the pre-intervention group (PrIG) in cycle 1 and the post-intervention group (PoIG) in cycle 2, each spanning a duration of two months. Ethical approval was sought prior to commencing this QIP from the Betsi Cadwaladr University Clinical Effectiveness Team and Ethical Committee with reference No.1312 dated April 26, 2023. Notably, there were no instances of follow-up loss observed within either of the study groups, underscoring the completeness of data collection and participant engagement throughout the investigation.

The inclusion criteria for this project were adult patients scheduled for elective vascular surgery. In this QIP, surgeries that were initially considered semi-elective, with a recommended start within 48 hours, have been reclassified as elective. Exclusion criteria were defined to exclude paediatric patients, pregnant women, adult patients scheduled for urgent or emergency vascular surgery, and patients who have diabetic gastroparesis, ensuring that the study's focus remained on the intended patient population. Patients in need of urgent or emergency vascular surgery are not included as they require immediate fasting following the surgical decision, leaving no opportunity for preoperative snack prescriptions.

Cycle 1: PrIG

A prospective analysis was conducted on a cohort of 20 patients scheduled for elective vascular surgical procedures. Patient data encompassed a range of variables including demographics, diabetic status, American Society of Anaesthesiologists (ASA) classification, chosen anaesthetic method, preoperative meal timing, i.e., the closest preoperative breakfast, lunch, or dinner, the time of anaesthesia commencement (in a 24-hour format), and the initiation of the surgical procedure (in a 24-hour format). Subsequently, the elapsed duration between the preoperative meal and both anaesthesia and surgical commencement were calculated. Additionally, patients were graciously invited to partake in a concise survey consisting of two inquiries. The first inquiry sought their valuable feedback on the existing hospital preoperative fasting protocol, while the second probed their inclinations regarding potential modifications to this established practice.

Intervention and quality improvement

Preoperative snack prescriptions, i.e. biscuits, chips, or cakes, were integrated into the official drug prescription chart, and administered at specific times tailored to each patient's scheduled surgery. Patients scheduled for morning operative lists (before noon) received preoperative snack prescriptions at 23:00 on the preceding day. In contrast, patients scheduled for evening operative lists (afternoon) were prescribed preoperative snacks to be administered at two distinct intervals: 23:00 on the day preceding surgery and 06:00 on the day of the surgical procedure.

Cycle 2: PoIG

A prospective analysis was conducted on a cohort of 20 elective surgical patients. Comprehensive patient data encompassed demographic information, diabetic status, ASA classification, chosen anaesthetic method, preoperative meal timings, and the timing of anaesthesia and surgical commencement, all recorded in a 24-hour format. The quality improvement introduced an intervention involving preoperative snack prescription at this cycle of patient care. Subsequently, the time intervals between preoperative snack prescription, anaesthesia, and surgical commencement were calculated. Furthermore, patients were invited to participate in a concise survey comprising two inquiries. The first inquiry aimed to gather the patients' valuable feedback regarding the introduced intervention, specifically focusing on preoperative snack prescription. The second inquiry sought insights into their preferences concerning potential modifications to this established practice.

Data analysis

All data were subjected to statistical analysis utilizing IBM SPSS Statistics for Windows, Version 26.0 (Released 2019; IBM Corp., Armonk, New York, United States), in conjunction with Microsoft Excel (Microsoft Corporation, Redmond, Washington, United States). 2022.

## Results

In Table 1, we present the demographic characteristics of two distinct patient cohorts: the PrIG and the PoIG. Within the PrIG, 40% of the 20 patients were female, with an average age of 74 years (standard deviation, 6.9; range, 61-86 years). 80% (16 out of 20) of the patients in this group opted for general anaesthesia (GA) as their preferred method of anaesthesia, while the remaining patients chose monitored anaesthesia care (MAC), i.e., local anaesthesia with sedation. In contrast, the PoIG comprised 35% female patients (seven out of 20) with a mean age of 61.3 years (standard deviation, 11.9; range, 36-81 years). Among this cohort, 60% (12 out of 20) chose GA as their anaesthesia method of choice.

**Table 1:** Demographic data of the patients. PrIG = Pre-intervention group; PoIG = Post-intervention group; M = Male; F = Female; NA = Non-diabetic; T1DM (I) = Type 1 diabetes mellitus on insulin therapy; T2DM (D) = Type 2 diabetes mellitus on diet control; T2DM (O) = Type 2 diabetes mellitus on oral hypoglycaemic medication; T2DM (I) = Type 2 diabetes mellitus on insulin therapy; GA = General anaesthesia; MAC = Monitored anaesthesia care, i.e., local anaesthesia with sedation; ASA: American Society of Anaesthesiologists

Patient	Gender	Age	ASA	Diabetic Status	Anaesthetic Method	Patient	Gender	Age	ASA	Diabetic Status	Anaesthetic Method
PrIG						PoIG					
1	M	82	2	T2DM (O)	MAC	1	F	69	3	T2DM (O)	GA
2	F	69	2	NA	GA	2	M	73	4	T2DM (O)	MAC
3	M	86	4	Pre-diabetic	GA	3	M	65	3	NA	GA
4	M	64	3	NA	GA	4	M	77	3	T2DM (O)	GA
5	M	72	3	NA	GA	5	M	70	3	T2DM (D)	GA
6	M	75	3	NA	GA	6	F	63	3	NA	MAC
7	M	61	3	NA	GA	7	M	69	3	T2DM (D)	GA
8	F	66	3	NA	GA	8	M	72	3	NA	GA
9	M	76	3	T2DM (D)	GA	9	M	36	3	NA	GA
10	M	82	2	T2DM (D)	GA	10	M	37	3	NA	GA
11	M	64	2	T2DM (I)	GA	11	M	54	3	T2DM (O)	GA
12	F	80	3	NA	GA	12	F	47	2	T2DM (I)	GA
13	M	75	2	T2DM (O)	GA	13	F	65	3	NA	MAC
14	F	78	3	T2DM (D)	GA	14	M	61	3	T2DM (I)	MAC
15	F	76	3	NA	MAC	15	F	55	4	T1DM (I)	MAC
16	F	73	3	NA	MAC	16	F	60	4	T1DM (I)	MAC
17	M	70	3	T2DM (O)	GA	17	F	60	4	T1DM (I)	GA
18	M	73	4	NA	GA	18	M	53	3	T2DM (I)	MAC
19	F	84	4	NA	MAC	19	M	59	4	T2DM (O)	GA
20	F	74	3	NA	GA	20	M	81	3	T2DM (O)	MAC

Tables 2-3 provide a comprehensive overview of crucial temporal parameters, including the timing of preoperative meals, the scheduling of preoperative snack prescriptions for the PoIG, and the corresponding durations between these events and both anaesthesia and surgery commencement.

**Table 2:** Timing of preoperative meals, anaesthesia commencement, surgical commencement, and the durations between these events for PrIG. PrIG = Pre-intervention group; Hr = Hours

Patient	Time of Preoperative Meal, 24-hour format	Time of Anaesthesia Commencement, 24-hour format	Time of Surgery Commencement, 24-hour format	Duration between Preoperative Meal and Anaesthesia Commencement, hr	Duration between Preoperative Meal and Surgery Commencement, hr
1	17:00	10:40	10:40	17.7	17.7
2	18:00	09:20	10:00	15.3	16.0
3	17:00	10:17	11:20	17.3	18.3
4	18:00	09:15	10:30	15.3	16.5
5	17:30	10:10	10:30	16.7	17.0
6	18:00	14:07	15:00	20.1	21.0
7	18:00	10:40	11:45	16.7	17.8
8	16:00	14:30	15:15	22.5	23.3
9	16:00	11:20	12:00	19.3	20.0
10	17:00	10:18	11:15	17.3	18.3
11	17:00	10:20	11:00	17.3	18.0
12	17:00	11:50	12:20	18.8	19.3
13	21:00	14:30	15:20	17.5	18.3
14	18:00	12:15	12:50	18.3	18.8
15	19:00	15:20	15:50	20.3	20.8
16	19:00	14:40	15:15	19.7	20.3
17	18:00	10:10	11:00	16.2	17.0
18	19:00	09:36	11:35	14.6	16.6
19	17:00	09:45	10:20	16.8	17.3
20	17:00	10:20	11:15	17.3	18.3

**Table 3:** Timing of preoperative meals, anaesthesia commencement, surgical commencement, and the durations between these events for PoIG. PoIG = Post-intervention group; Hr = Hours

Patient	Time of Preoperative Meal, 24-hour format	Time of Preoperative Snack Prescription, 24-hour format	Time of Anaesthesia Commencement, 24-hour format	Time of Surgery Commencement,24-hour format	Duration between Preoperative Meal and Anaesthesia Commencement, hr	Duration between Preoperative Meal and Surgery Commencement, hr	Duration between Preoperative Snack Prescription and Anaesthesia Commencement, hr	Duration between Preoperative Snack Prescription and Surgery Commencement, hr
1	20:30	23:00	09:15	11:10	12.8	14.7	10.3	12.2
2	17:00	06:30	13:00	14:00	20.0	21.0	6.5	7.5
3	17:00	23:00	09:30	10:30	16.5	17.5	10.5	11.5
4	17:30	23:00	12:50	14:45	19.3	21.3	13.8	15.8
5	18:00	06:00	13:30	14:40	19.5	20.7	7.5	8.7
6	17:00	23:00	09:45	11:45	16.8	18.8	10.8	12.8
7	18:00	23:30	10:00	10:50	16.0	16.8	10.5	11.3
8	18:00	23:00	09:50	11:50	15.8	17.8	10.8	12.8
9	18:00	23:00	09:40	10:30	15.7	16.5	10.7	11.5
10	18:00	06:00	14:10	14:30	20.2	20.5	8.2	8.5
11	17:00	06:00	14:20	15:00	21.3	22.0	8.3	9.0
12	18:00	23:00	09:45	10:00	15.8	16.0	10.8	11.0
13	17:30	23:00	11:30	12:20	18.0	18.8	12.5	13.3
14	17:00	23:00	13:10	14:10	20.0	21.2	14.0	15.2
15	17:00	23:00	14:00	14:40	21.0	20.7	15.0	15.7
16	17:00	23:00	15:00	15:30	22.0	22.5	16.0	16.5
17	17:30	23:00	14:25	15:20	20.9	21.8	15.4	16.3
18	17:00	06:00	14:40	15:30	21.7	22.5	8.7	9.5
19	17:30	06:00	15:00	16:40	21.5	23.2	9.0	10.7
20	17:30	06:00	14:40	15:40	21.2	22.2	8.7	9.7

In the PrIG, the mean duration from the preoperative meal to anaesthesia commencement was 17.8 hours (standard deviation, 1.9; range, 14.6-22.5 hours). Similarly, the mean duration from the preoperative meal to the initiation of surgery was 18.5 hours (standard deviation, 1.8; range, 16.0-23.3 hours).

In the PoIG, the mean duration between the preoperative meal and anaesthesia commencement was 18.8 hours (standard deviation, 2.6; range, 12.8-22.0 hours), with the mean duration from the preoperative meal to surgery commencement measuring 19.8 hours (standard deviation, 2.5; range, 14.7-23.2 hours). However, the mean time interval between preoperative snack prescription and anaesthesia commencement was 10.9 hours (standard deviation, 2.7; range, 6.5-16.0 hours), while the mean duration from preoperative snack prescription to surgery commencement was 12.0 hours (standard deviation, 2.8; range, 7.5-16.5 hours).

A noteworthy disparity emerges when comparing the PrIG and the PoIG. Specifically, there is a significant difference in the mean time intervals between the patient's last food intake, i.e., preoperative meal for PrIG or the preoperative snack for PoIG, and the subsequent events of anaesthesia commencement. This difference was statistically significant, with a mean time difference of 6.9 hours (95% confidence interval, 5.3-8.4 hours; p < 0.001).

Similarly, when examining the time elapsed between the last food intake and surgery commencement, we observe a notable mean time difference between PrIG and PoIG, amounting to 6.6 hours (95% confidence interval, 5.0-8.1 hours; p < 0.001). These statistically significant differences underscore the profound impact of the intervention on the timing of key preoperative events in both patient groups.

In Table 4, we turn our attention to patient satisfaction regarding the preoperative fasting protocol and their expressed desire for improvement. In the PrIG, a striking 95% of patients (19 out of 20) reported dissatisfaction with the existing hospital practice of preoperative fasting, and notably, all patients (20 out of 20) expressed a strong desire for improvement.

**Table 4:** Patients’ satisfaction. PrIG = Pre-intervention group; PoIG = Post-intervention group

Patient	Satisfaction for Pre-intervention Preoperative Fasting	Advocate for Improvement	Patient	Satisfaction for Post-intervention Preoperative Fasting	Advocate for Improvement
PrIG			PoIG		
1	No	Yes	1	Yes	No
2	No	Yes	2	Yes	No
3	No	Yes	3	Yes	No
4	No	Yes	4	Yes	No
5	No	Yes	5	Yes	No
6	No	Yes	6	Yes	No
7	Yes	Yes	7	Yes	No
8	No	Yes	8	Yes	No
9	No	Yes	9	Yes	No
10	No	Yes	10	Yes	No
11	No	Yes	11	Yes	No
12	No	Yes	12	Yes	No
13	No	Yes	13	Yes	No
14	No	Yes	14	Yes	No
15	No	Yes	15	Yes	No
16	No	Yes	16	Yes	No
17	No	Yes	17	Yes	No
18	No	Yes	18	Yes	No
19	No	Yes	19	Yes	No
20	No	Yes	20	Yes	No

Conversely, in the PoIG, none of the patients expressed dissatisfaction with the intervention. This intervention involved the practice of preoperative snack prescription, strategically designed to bridge the temporal gap between the preoperative meal and the scheduled surgical procedure. Furthermore, none of the patients in the PoIG indicated a wish for further improvement in the preoperative fasting regimen, signalling a high level of satisfaction with the implemented preoperative snack prescription. These findings underscore the potential of preoperative snack prescription as a valuable tool in optimizing patient satisfaction and addressing longstanding concerns related to preoperative fasting protocols.

## Discussion

The utilization of preoperative fasting guidelines is a common practice among healthcare professionals in the United Kingdom, with the National Institute for Health and Care (NICE) guidelines serving as a central reference point. NICE guidelines, developed by independent committees comprising professionals and lay members and informed by stakeholder consultations, offer evidence-based recommendations for various aspects of healthcare. In line with NICE guideline NG180, which conducted an evidence review on preoperative fasting, the recommended fasting time falls within the range of four to six hours [4]. Furthermore, the European Society of Anaesthesiology and Intensive Care (ESAIC) in 2011 reinforced the importance of a minimum preoperative fasting time of six hours [5]. According to the 2017 guidelines of the ASA, it is recommended that a preoperative fasting period of six hours be observed for light meals, while heavy meals, including fried or fatty foods, and meat, may require an extended preoperative fasting period [6]. In our QIP, we closely align with these guidelines.

Without the inclusion of preoperative snack prescription, our investigation revealed that for both the PrIG and the PoIG, the average preoperative fasting times before anaesthesia and surgery commencement were 18.3 hours (standard deviation, 2.4; range, 12.8-22.5 hours) and 19.2 hours (standard deviation, 2.3; range, 14.7-23.3 hours), respectively. When comparing these findings with the recommended guidelines, which advocate for a preoperative fasting time of six hours, our QIP unveiled an additional 12.3 hours and 13.2 hours of preoperative fasting time before anaesthesia and surgery commencement, respectively.

Our project results are consistent with the findings of De Aguilar-Nascimento et al., who conducted the BIGFAST multiple study involving 3,715 patients from 16 Brazilian hospitals. They reported a median preoperative fasting time of 12 hours (range, 2-216 hours) and highlighted that nearly 80% of patients were operated on after fasting for eight hours or more, with 46.2% fasting for over 12 hours, which was more prominent in hospitals following traditional fasting protocols [7]. Similarly, Cestonaro et al. reported a median preoperative fasting time of 16.5 hours (range, 5.5-59.92 hours) in a study involving 135 patients conducted in 2014 [8].

A cross-sectional study by Francisco et al. involving 65 patients undergoing abdominal elective surgery found a real fasting average time of 16 hours (range, 9.5-41.6 hours), which exceeded the intended fasting time of 11 hours (range, 6.6-26.8 hours) [9]. Segaran et al. conducted a web-based survey involving 232 United Kingdom ICUs, revealing variations in fasting times for different surgical procedures and highlighting the national inconsistency in fasting practices, which could potentially lead to suboptimal enteral nutrition delivery [10].

In response to the observed divergence in fasting practices, our QIP proposed the implementation of preoperative snack prescriptions in the patient's official drug prescription chart. This approach aimed to ensure that preoperative snacks were administered at specific, desirable times, aligning with the guidelines while avoiding the risk of complications such as aspiration due to shortened fasting periods. As a result of preoperative snack prescription, the average time interval between preoperative snack prescription and anaesthesia commencement was reduced to 10.9 hours (standard deviation, 2.7; range, 6.5-16.0 hours), while the average duration from preoperative snack prescription to surgery commencement was 12.0 hours (standard deviation, 2.8; range, 7.5-16.5 hours). This effectively narrowed the gap between preoperative fasting times in our study and the guidelines to 4.9 hours and 6.0 hours for anaesthesia and surgery commencement, respectively. Although our project did not achieve complete alignment with the guidelines regarding hospital preoperative fasting practices, it did demonstrate a significant improvement in patient satisfaction with the intervention, as illustrated in Table 3.

Gonik et al. conducted a randomized controlled trial assessing the feasibility of shortened fasts in intubated ICU patients undergoing tracheotomy. The study included 24 patients allocated to two preoperative fasting regimens: a six-hour fast as the control group and a 45-minute fast as the intervention group. They reported no cases of intra-operative aspiration and a single case of postoperative pneumonia in the intervention group. The median of fast and caloric delivery significantly differed between the control group and the shortened fast group: 22 hours vs. 14 hours and 429 kcal vs. 1050 kcal, respectively [11].

However, the classification of milk as either solid food or liquid has been a subject of debate. Many preoperative fasting guidelines suggest that hot tea or coffee with added milk should be considered similar to solid food, requiring an interval of six hours before anaesthesia [12-14]. Hillyard et al. conducted a randomized controlled crossover study involving 10 healthy volunteers in 2014, utilizing paracetamol absorption techniques and real-time ultrasound measurements of the gastric antrum's cross-sectional area to study gastric emptying after the ingestion of tea with milk. The study found no significant difference in gastric emptying times when a modest amount of milk was added to tea [15].

Bang et al. also advocated the use of chewing gum during preoperative fasting until surgery commencement for patient-centred outcomes, another topic mentioned in the ESAIC 2011 guidelines [5,16]. In their study involving 94 patients, they reported lower preoperative anxiety levels in the intervention group that used chewing gum and no significant difference in gastric fluid analysis compared to the control group. There was also no increased risk of pulmonary aspiration in the intervention group [16].

While there is limited research on preoperative solid snack prescription, multiple studies have explored the effect of preoperative oral carbohydrate (CHO) administration. Shi et al. randomized 75 patients into three groups: those who received preoperative CHO, those who received flavoured water, and a control group without any prescription. Patients who received CHO and flavoured drinks did not exhibit increased gastric fluid volumes, and there were no adverse events compared to the control group. CHO administration also reduced the sense of thirst, hunger, and anxiety among patients [17]. Itou et al. reported insignificant differences in mean gastric fluid volume and pH between patients who received preoperative carbohydrate drinks and those who did not [18]. This evidence is supported by studies conducted by Yagci et al. and Choi et al., both concluding that gastric volume is not affected by carbohydrate drinks [19-20].

Melis et al. conducted a study where they demonstrated that preoperative CHO administration has the potential to prevent surgery-induced immunodepression, offering a promising avenue to reduce the risk of infectious complications associated with surgery [21]. In a study by Hausel et al., pre-operative CHO intake was found to effectively reduce the incidence of postoperative nausea and vomiting [22]. Moreover, another investigation by Hausel et al. concluded that CHO intake did not result in an increase in gastric fluid volume or affect gastric acidity. This intervention was also associated with reduced discomfort during the preoperative waiting period for elective surgery [23].

Patient comfort and satisfaction were notable outcomes of CHO administration, as revealed in the study conducted by Bopp et al. [24]. Additionally, Wang et al. reported that the oral administration of preoperative CHO had a positive impact on patients' sensations of thirst, hunger, and mouth dryness during the postoperative period [25]. These findings align with the results presented by Meisner et al. [26].

Furthermore, De Carvalho et al. took a step further by enhancing CHO intake with the addition of whey protein (CHO-P) in their study [27]. Their research indicated that patients who received CHO-P experienced a lower risk of postoperative complications compared to those who received CHO alone, underscoring the potential benefits of this approach in optimizing surgical outcomes.

Limitations

This QIP acknowledges certain limitations that warrant consideration. Firstly, it is important to recognize the constraint posed by a relatively small sample size within our QIP. This limitation may affect the generalizability of our findings to a broader patient population. Additionally, our QIP did not encompass the assessment of gastric pH and volume following preoperative snack prescription, in contrast to some studies discussed in the paper. This decision was made in adherence to the guidelines outlined by the ESAIC in 2011, the NICE guidelines, and the ASA 2017 guidelines, which guided the scope of our QIP. Furthermore, due to the alignment of our preoperative snack prescription timing with recommended guidelines, it became challenging to assess peri-operative aspiration rates specifically attributable to preoperative snack prescription. Lastly, while we aimed to scrutinise potential complications associated with preoperative snack prescription, the comprehensive evaluation of all conceivable complications remains challenging given the multifactorial nature of peri-operative care. These limitations underscore the need for future research with larger sample sizes and a broader scope to provide a more comprehensive understanding of the topic at hand.

## Conclusions

Our QIP has introduced the concept of preoperative snack prescription into the local hospital's preoperative care policy, with a strong emphasis on striking a balance between patient safety and comfort. As a single-centre experience, we observed that the average time between preoperative fasting without a preoperative snack and the commencement of anaesthesia for both PrIG and PoIG was 18.3 hours. Following the implementation of preoperative snacks, this fasting time was reduced to 10.9 hours before anaesthesia commencement.

Our study bears relevance to healthcare professionals and underscores the advantages of shortened fasting periods, leading to enhanced patient satisfaction and comfort. We strongly advocate for the adoption of this approach to improve peri-operative care, align with evidence-based practices, and foster a more compassionate healthcare environment. This QIP contributes to the ongoing evolution of surgical medicine, ultimately enhancing patient safety, well-being, and the overall healthcare experience.
